# Modified technique of extraperitoneal colostomy without incision of the posterior rectus sheath

**DOI:** 10.1038/s41598-021-82626-1

**Published:** 2021-02-03

**Authors:** Tao Zhang, Daye Yang, Gongping Sun, Dewei Zhang

**Affiliations:** Department of Colorectal and Hernia Surgery, The Fourth Affiliated Hospital of China Medical University, 4# Chong-shan East Road, Shenyang, Liaoning China

**Keywords:** Cancer, Gastroenterology

## Abstract

Extraperitoneal colostomy is a widely used technique during abdominoperineal resection (APR) operation for lower rectal cancer. This technique has been reported to be effective to prevent the postoperative parastomal hernia in some retrospective studies, however, there is still a certain incidence of parastomal hernia. A modification of the extraperitoneal colostomy technique is described in this paper that keeps posterior rectal sheath intact instead of having a conventional incision, to further reduce the risk of parastomal hernia. Until now, this modified technique has been performed in 15 patients, no occurrence of parastomal hernia was observed.

## Introduction

In the curative treatment of low rectal cancer, approximately 10–20% of patients need the abdominoperineal resection (APR) along with a permanent colostomy^1^. The existence of stoma mainly affects the quality of life of patients. Of all the stoma-related complications, parastomal hernia is one of the most common and troublesome complications after sigmoid colostomy. It is reported that the traditional intraperitoneal method of sigmoid colostomy has a 4–48.1% incidence of parastomal hernia^2–4^. One method to prevent parastomal hernia is the utilization of prophylactic synthetic nonabsorbable meshes which are suggested by the latest European Hernia Society guidelines^5^. Another method to reduce the incidence of parastomal hernia is the extraperitoneal route for stoma construction that was firstly proposed by Goligher^6^. Meta-analysis indicates this technique of extraperitoneal colostomy significantly lowers the incidence of parastomal hernia^7,8^. However, the non-randomized design of the included studies limits its certainty.

Therefore, we as surgeons, still continue to make efforts to improve the technique of extraperitoneal colostomy, here, we introduced a modified technique to make the stoma without damaging the posterior sheath of rectus abdominis.

## Methods

**Figure 1:**
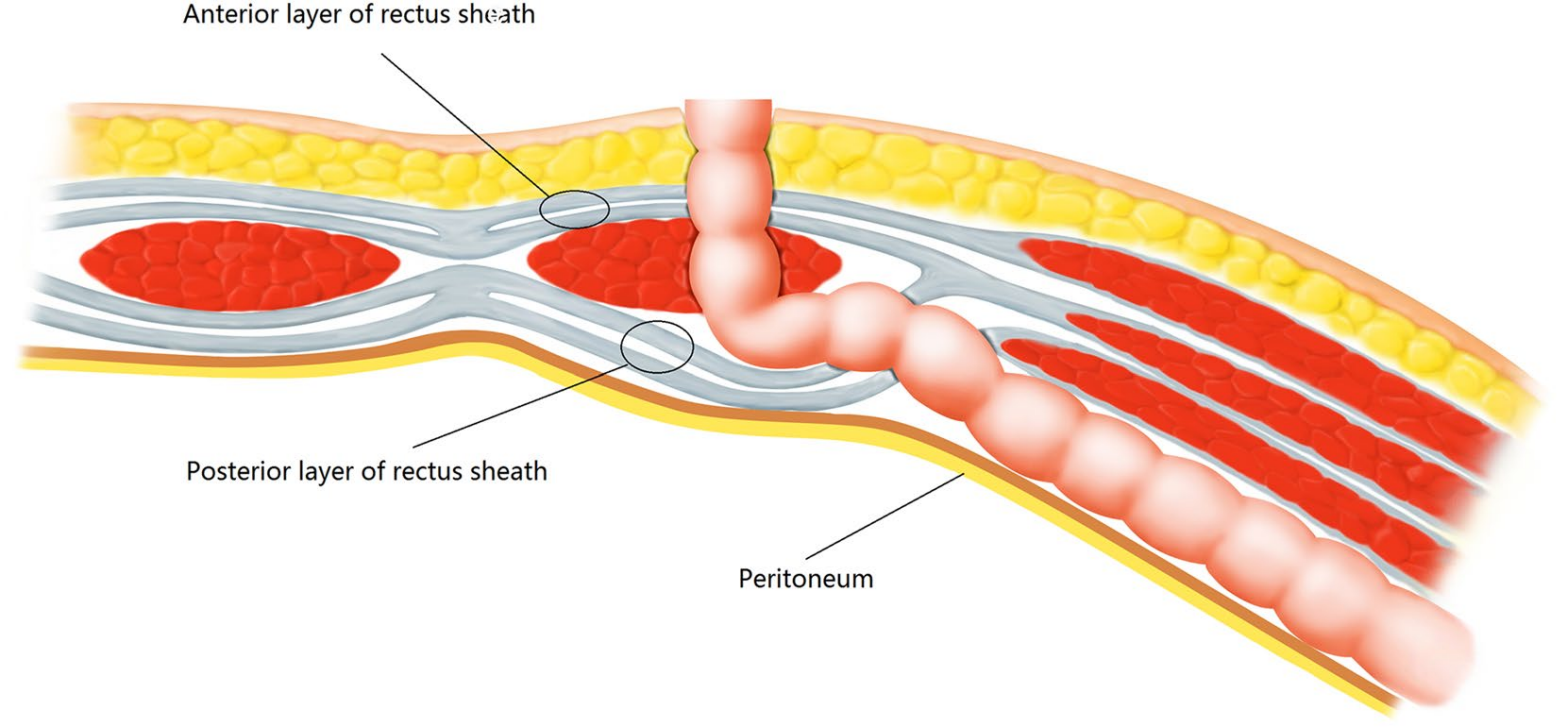
The cross-section diagram of abdominal wall structure during extraperitoneal colostomy.

**Figure 2:**
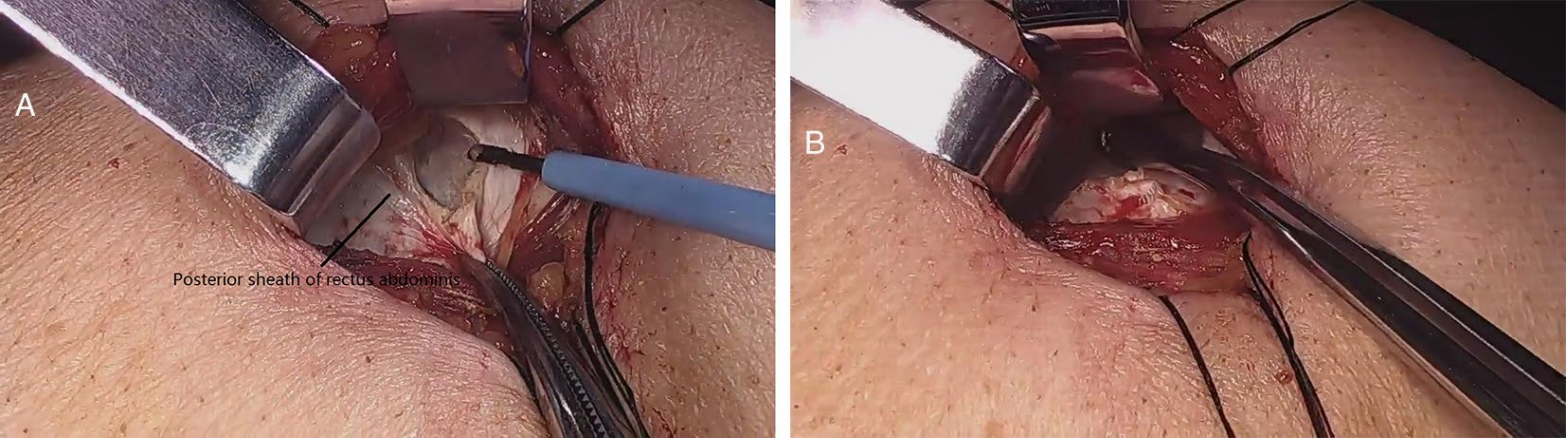
The transversus abdominis muscle was release from the lateral edge of the posterior rectal sheath (**A**) and the sheath was opened about 6 cm in width (**B**).

**Figure 3:**
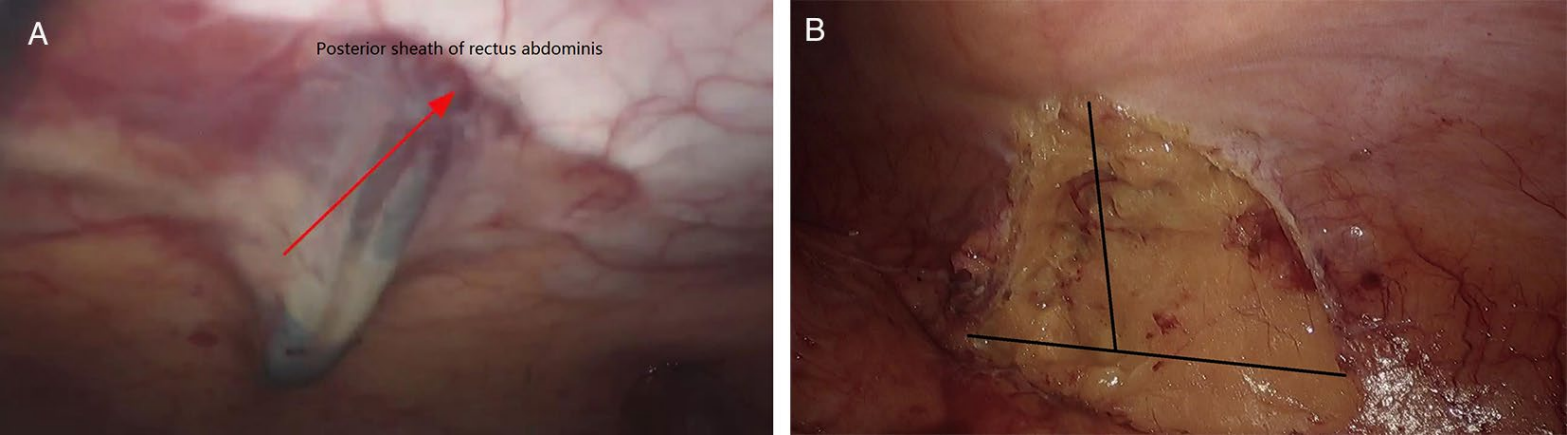
The extraperitoneal space was separated by a pair of curved Kelly forceps (**A**) and the peritoneum inside the abdominal cavity is opened in a reverse “T” type (**B**).

**Figure 4:**
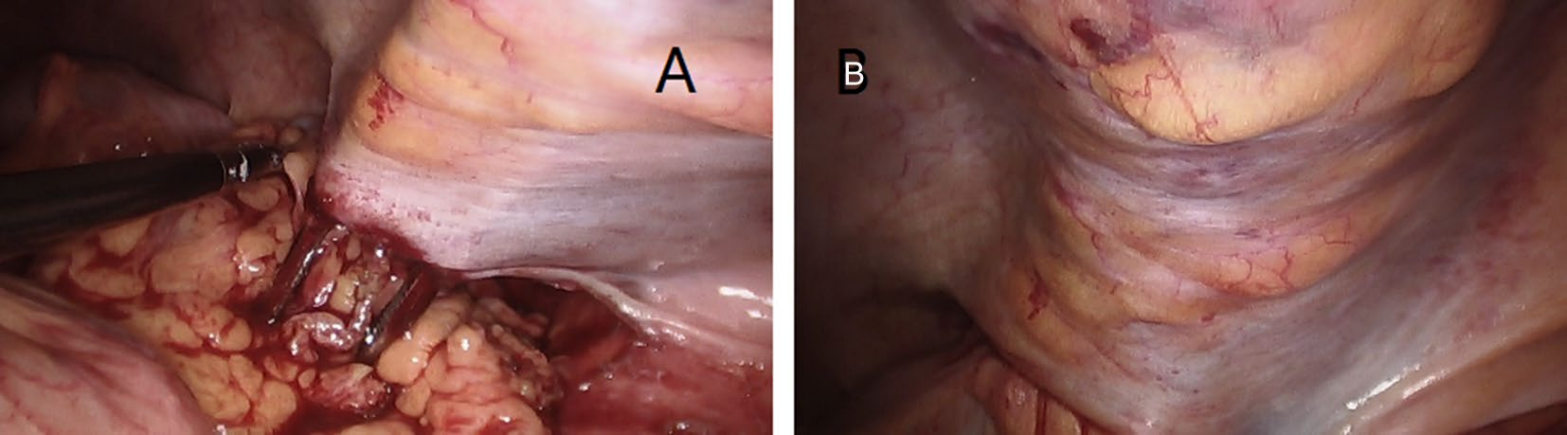
The tip of Kelly forceps has grasped the stump of the sigmoid colon (**A**) and drawn through the tunnel (**B**).

The site of colostomy was marked preoperatively by the stoma therapist. Whether the enterostomy should be placed through, or lateral to the rectus abdominis muscle to prevent parastomal herniation is still unclear^9^, the site we chose was located just across over the left rectus abdominis muscle at or slightly above the level of the umbilicus. After completion of APR, the operation of abdominal part was performed (Fig. 1). At the pre-marked stoma site, the skin was cut about 2.5–3 cm in diameter, the subcutaneous fat tissue was pushed aside, and then a cruciate incision was performed in the anterior rectal sheath. The abdominal rectus muscle was split about 2-finger width with forceps and retractor to expose the posterior rectal sheath. Here, the space was expanded to the lateral edge of the posterior sheath without damaging the sheath, the transversus abdominis muscle was released from the lateral edge of the posterior sheath about 6 cm in width (Fig. 2). The extraperitoneal space was bluntly separated by a pair of curved Kelly forceps just between the transversalis fascias and the transverse muscles, so the eatraperitoneal tunnel was created. The tip of the forceps followed this extraperitoneal tunnel and made the tunnel to a greater extent, the dissecting position of the peritoneum inside of the abdominal cavity was confirmed by direct observation (Fig. 3A). A reverse T-type incision of peritoneum was performed to allow the colon to pass smoothly (Fig. 3B). At last, the stump of the sigmoid colon was pulled out through the tunnel (Fig. 4).

The study was performed in accordance with the guidelines for good clinical practice. This study was approved by the Hospital Ethics Committee of China Medical University and informed consent was obtained from each participating patient.

## Results

All surgeries were performed by a single surgeon (D.Z.). We have applied this technique for 15 cases of lower rectal cancer from 2017 to 2019. Six cases were males and 9 cases were females. Age was 76-year old (61–85). The average BMI was 21.1 ± 2.3 (means ± SD).The mean operation time was 240 ± 19 min (means ± SD). (Table 1.) For extraperitoneal colostomy, the main complication is bleeding during the process of extraperitoneal channel formation. There were no such and other surgical complications during the operation. In one patient, at the early time after operation, the stoma obstruction was observed but there was no effect on exhaust, this was released by finger dilation of the stoma.

**Table 1:** Characteristics of patients and study informations.

Age	Gender	BMI (kg/m^2^)	Operation time (min)	Follow-up period (d)
77	M	21.7	240	619
61	F	19.1	245	582
65	F	18.7	240	485
76	F	22.1	210	410
76	M	21.2	270	365
66	M	20.1	235	330
80	F	19.1	240	317
63	M	23.2	260	275
78	F	26.5	220	255
75	F	21.1	255	230
69	M	18.3	270	212
85	F	22.3	240	195
78	M	21.2	215	167
70	F	18.5	250	155
79	F	19.6	210	120
76		21.1	240	275

The diagnosis of parastomal hernia was confirmed by both physical and/or radiological examination. Clinical diagnosis was done with the patient standing position and computed tomography (CT) was scanned in supine position meanwhile performing a valsalva manoeuvre. The patients return to our hospital every three months for a check. The follow-up period was calculated from the date of surgery to the latest CT scan. During the follow-up period, all patients received at least one CT scan. No parastomal hernia, either clinical or radiological, were observed in the follow-up period 275 ± 184 (median,IQR) postoperative days.

## Discussion

With the increasing incidences of rectal cancer in China, the surgical technique of treatment is also improving. Either open or laparoscopic APR for low rectal cancer can be chosen, permanent sigmoid colostomy is always an important component in these two kinds of APR. The creation of stoma seems easy to a surgeon, but when it is performed badly, patients may suffered from complications such as leakage, prolapse, parastomal hernias, retraction and stenosis. Parastomal hernia is one of the most common complications that can cause problems ranging from parastomal discomfort or pain to life-threatening complications, such as strangulation, perforation and obstruction^2^. The result of surgical treatment is not very satisfactory, and the recurrence rate is high. At present, the recurrence rate of Sugarbaker and Keyhole technique is still 11.6–34.6%^10^. Therefore, prevention is more important than treatment.

Many factors, such as the site or size of the stoma, and the fixation of the facia, were proven to have no effect on the formation of parastomal hernia^9^. Until now, the widely used techniques to prevent the hernia formation, including preventive mesh placement and extraperitoneal route for stoma construction, were proven to be effective^11,12^. The utilization of prophylactic meshes for end colostomies is strongly recommended by the European Hernia Society (EHS). However, such technique may require longer operative time and the high cost should also be taken into consideration. The EHS also suggests auditing the clinical outcomes and reporting the adverse events.

Although several studies have reported a lower rate of parastomal hernia with extraperitoneal colostomy compared to transperitoneal route^12,13^, all these studies are non-randomized analysis, further randomized controlled trials (RCT) are needed to confirm whether the extraperitoneal route could prevent parastomal hernia. The extraperitoneal technique is technically difficult to perform than transperitoneal colostomy. The longer extraperitoneal route may require to mobilize the splenic flexure. All of those may need more operative time, and that retraction and necrosis might even occur more frequently^14^. However, we did not encounter these difficulties, all the cases did not need the splenic flexure mobilization and there were no complications such as stoma retraction or necrosis. On the other hand, the traditional extraperitoneal colostomy is still at risk of parastomal hernia and has a certain incidence (3.5–6.4%)^4,7^. Here we introduced a modified technique that differs from the traditional methods, i.e. keeping the intact of the posterior rectal sheath instead of having a conventional incision.

It is reported that the main reason for the occurrence of the parastomal hernia is the weakness between the colon and the abdominal wall^15^. The current technique reinforces the space between colon and the abdominal wall, although one patient suffered from incomplete stoma obstruction, it was easily resolved and had no adverse effect on patient. We have proven the feasibility and surgical effectiveness of this practice. In our study, the shortest follow-up time is about 3 months, it is theoretically short but the parastomal hernia may already happen^16^, in addition, most hernias develop in the first few years, especially in the first year^2^. So we think the follow-up time in this paper is effective.

## Conclusions

The modified technique of the extraperitoneal colostomy which is described in this paper that keeps posterior rectal sheath intact instead of having a conventional incision, is effective to prevent the development of parastomal hernia. The technique is very promising but needs further evaluation with more patient cases over a longer follow-up period.
